# Morphological Traits and Water–Nutrient Utilization Efficiency of *Hippophae rhamnoides* Fine Roots Under Different Stubble Heights in Arsenic Sandstone Area, Inner Mongolia

**DOI:** 10.3390/plants14091329

**Published:** 2025-04-28

**Authors:** Yajie Xu, Yuefeng Guo, Yongjie Yue, Longfei Hao, Wei Qi, Runhong Gao, Xiaoyu Dong

**Affiliations:** 1 College of Forestry, Inner Mongolia Agricultural University, Hohhot 010010, Chinawolongyue123@163.com (Y.Y.); haolongfei_00@126.com (L.H.); 2 College of Desert Control Science and Engineering, Inner Mongolia Agricultural University, Hohhot 010010, China; 13684730161@163.com; 3 Inner Mongolia Autonomous Region Water Conservancy Development Center, Hohhot 010020, China; qiwemail@126.com

**Keywords:** arsenic sandstone area, stubble height, fine root morphological traits, phosphorus limitation

## Abstract

*Hippophae rhamnoides* (family Elaeagnaceae) is a deciduous shrub that has become a uniquely advantageous species in the arsenic sandstone area of Inner Mongolia due to its well-developed root system and strong tillering ability. This study, by taking 10-year-old *H. rhamnoides* in the arsenic sandstone area as the research object and analyzing the morphological traits of their fine roots and their coordination within soil under different stubble heights (0, 10, 15, and 20 cm) and non-stubble treatment, aims to select the optimal stubble height that is most conducive to the rejuvenation of *H. rhamnoides* and thus improve the decline in the productivity of *H. rhamnoides* in this region. The results reveal significant differences in fine root and soil properties under different stubble heights (*p* < 0.05). Among different traits, fine root area density shows the highest total coefficient of variation, making it the most sensitive trait. Principal component analysis results indicate that after stubble treatment, the traits of *H. rhamnoides* fine roots center on high specific surface area (0.316) + high specific root length (0.312), shifting toward a resource-acquisition ecological strategy with the best foraging efficiency observed under a stubble height of 15 cm. Soil N:P and C:P can explain 66% and 61% of the root morphological traits strategies deployed during stubble treatment, respectively. Fine roots exhibit high adaptability to the breaking of phosphorus limitation and fixation of carbon and nitrogen.

## 1. Introduction

Arsenic sandstone refers to interbedded rock layers composed of thick sandstone, sandy shale, and argillaceous rocks from the Paleozoic Permian, Mesozoic Triassic, Jurassic, and Cretaceous periods [1]. It is mainly distributed in the junction of Shanxi, Shaanxi, and Inner Mongolia in the Yellow River Basin of China and a major source of coarse sediment in the middle reaches of the Yellow River. Given its unique mineral composition, arsenic sandstone has a low degree of lithification, making it highly susceptible to erosion and sediment production [2,3,4]. It has the characteristics of “becoming mud when mixed with water, and becoming sand when there is wind”. As a result, the arsenic sandstone area in Inner Mongolia is one of the regions with the most severe soil erosion on the Loess Plateau and even worldwide [5,6]. Artificial vegetation restoration is an important measure for ecological restoration in this region [7,8]. *Hippophae rhamnoides* (family Elaeagnaceae), with its well-developed root system, strong tillering and sprouting abilities, rapid reproduction, and high biomass, plays an important role in soil and water conservation [9]. It is a uniquely advantageous plant in the arsenic sandstone area of Inner Mongolia [10]. However, due to the special soil properties, specific geological conditions, and arid environment of the region, large areas of approximately 10-year-old *H. rhamnoides* forests experienced growth decline and reduced productivity [11,12]. Stubble treatment is a common forest management practice used for tree regeneration and rejuvenation. It restores and promotes growth; alters plant resource absorption and utilization strategies; rapidly restores population size, structure, and function; and ensures vegetation succession with a sustained niche effect, maintaining the dominant position of populations during succession [13]. Fine root morphological traits are an important indicator for studying the response and adaptation mechanisms of species, communities, and ecosystems to management measures, playing an important role in vegetation restoration.

Fine roots (typically defined as roots with a diameter ≤ 2 mm) are the primary drivers of carbon, nutrient, and water cycling in plants and ecosystems [14,15], accounting for 10–50% of annual net primary production [16,17]. As key organs for material exchange and energy transfer between plants and soil, fine roots exhibit high plasticity and extreme sensitivity to environmental changes. Their morphological and structural characteristics play a crucial role in driving a series of physiological and ecological functions at levels ranging from individual plants to ecosystems [18,19]. The morphological traits of fine roots are not only influenced by genetic characteristics, they also adapt to changes in soil environments and the needs of their own growth and development, reflecting the adaptive strategies of plants in their living environments [20,21]. On the basis of research on fine root traits, scholars have proposed a one-dimensional root economics spectrum (RES). On one end of the RES are plants with a conservative strategy. Such plants are characterized by thick roots, high root tissue density, and strong resource conservation capabilities. On the other end of the RES are plants with an acquisitive strategy. Such plants feature fine roots, high specific root length (SRL), and high resource acquisition efficiency [22]. Studies have shown that plants can adapt to external environments through various strategies, such as increasing their fine root biomass (RB), expanding their absorption area, adjusting their fine root distribution, or altering the morphological and physiological traits of their fine roots [23]. The root ecological strategy employed by plants depends on climate, species, and management practices [24]. When the aboveground parts of plants are damaged, the root system adjusts its morphological characteristics to adapt to the external environment. Research has demonstrated that stubble treatment can effectively enhance root stress resistance [25], promote fine root morphological traits, and support the compensatory growth of aboveground parts, thus achieving the goal of regeneration and rejuvenation [26]. Zheng [27] indicates that sprouting plants in arid regions rapidly increase their fine RB through compensatory growth after stubble treatment, improving the ability of tree fine roots to absorb water and nutrients, thereby quickly restoring aboveground productivity. Liu [28] subjected *H. rhamnoides* in the Loess Plateau of Shaanxi to 0, 10, and 20 cm stubble treatments. Their results showed that stubble treatment increased the distribution of nutrient elements in fine roots, with the 10 cm treatment being the most effective. Liu [29] studied the fine root and leaf functional traits of *H. rhamnoides* in a feldspathic sandstone area under treatment with different stubble heights. They discovered that stumped *H. rhamnoides* adopted a rapid investment–return-type resource trade-off strategy, and its growth rate was maximized under the stump height of 15 cm.

Research on the stubble treatment of *H. rhamnoides* in arsenic sandstone areas primarily focuses on aboveground parts, with aboveground functional traits serving as the main mechanism for explaining environmental and anthropogenic disturbances. Given the heterogeneity of belowground soil environments and limitations of research techniques, studies on belowground functional traits have lagged behind those on aboveground functional traits, leading to an asymmetry between aboveground and belowground research. This situation has severely constrained a deep understanding of plant adaptability to environmental conditions. Understanding how stubble treatment alters belowground ecological processes can not only clarify the driving factors and ecological strategies of fine root compensatory growth but can also help elucidate the synergistic interactions between plants and soil environments. Such an understanding is crucial for vegetation restoration and sustainable development in arsenic sandstone areas. Therefore, this study focuses on 10-year-old *H. rhamnoides* plantations in an arsenic sandstone area, analyzing fine root morphological traits and soil physicochemical properties under treatment with different stubble heights. Its objectives are (1) to reveal the fine root morphological trait strategies of *H. rhamnoides* after stubble treatment; (2) to explore the water and nutrient utilization efficiency of fine roots after stubble treatment; (3) and to select the stubble height that is most conducive to the regeneration and rejuvenation of *H. rhamnoides*.

## 2. Results

### 2.1. Effects of Stubble Treatment on the Fine Root Morphological Traits of H. rhamnoides

**Figure 1:**
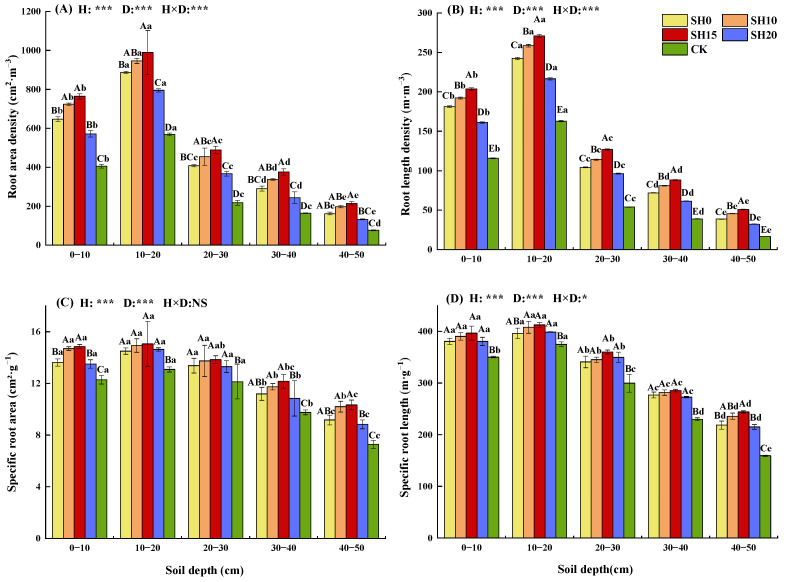
Effects of different stubble heights on the morphological characteristics of *H. rhamnoides* fine roots (mean ± SE). (**A**) Root area density; (**B**) root length density; (**C**) specific root area; (**D**) specific root length. SH0: stubble treatment at 0 cm above ground level, SH10: stubble treatment at 10 cm above ground level, SH15: stubble treatment at 15 cm above ground level, SH20: stubble treatment at 20 cm above ground level, and CK: non-stubble treatment. Different capital letters indicate significant differences among different stubble patterns in the same soil layer (*p* < 0.05), whereas different lowercase letters indicate significant differences between different soil layers in the same stubble patterns (*p* < 0.05). *: *p* < 0.05, ***: *p* < 0.001, NS: not significant.

Two-way ANOVA showed that stubble treatment highly significantly increased the RB, RLD, root area density (RAD), SRL, and specific surface area (SRA) of *H. rhamnoides* (*p* < 0.001) within the 0–50 cm soil layer (Figure 1, Table 1). The coefficient of variation (CV) for fine roots under different stubble treatments ranged from 6.36% to 21.64%, with the CV of RB, RLD, and RAD all exceeding 10% (Table 2). Highly significant differences in fine root traits among different soil layers (*p* < 0.001) were found, with the CV for fine roots in each layer ranging from 15% to 58.17% and exceeding 10%, indicating substantial variation in fine root distribution across soil layers. The interaction effect had a highly significant effect on RB, RLD, and RAD (*p* < 0.001); a significant effect on SRL (*p* < 0.05); and no significant effect on SRA (*p* > 0.05).

The fine roots of *H. rhamnoides* were widely distributed in the topsoil layer (0–20 cm), accounting for 60.00% ± 1.87% of the total fine RB in the 0–50 cm soil layer (Table 1). As soil depth increased, fine root morphological traits first increased and then decreased, reaching their maximum values at the 0–20 cm soil layer (*p* < 0.05). The decline in SRL and SRA was slower compared to other traits. Stubble treatment significantly increased RB, RLD, RAD, SRL, and SRA (*p* < 0.05), with RAD showing the most pronounced increase, Specifically, RAD under stubble treatment was 1.75 ± 0.19 times higher than that under CK. Fine root morphological traits peaked under SH15 and then under SH10. Stubble treatment significantly increased the proportion of fine root in the 30–50 cm soil layer, with RLD showing the greatest increase among traits: RLD under stubble treatment increased by 4.51% ± 0.09% relative to that under CK. These results indicate that stubble treatment significantly promoted fine root growth, enhanced the ability of fine roots to absorb water and nutrients, and induced a tendency for the expansion of fine roots into deep soil.

### 2.2. Effects of Stubble Treatments on Soil Properties

**Figure 2:**
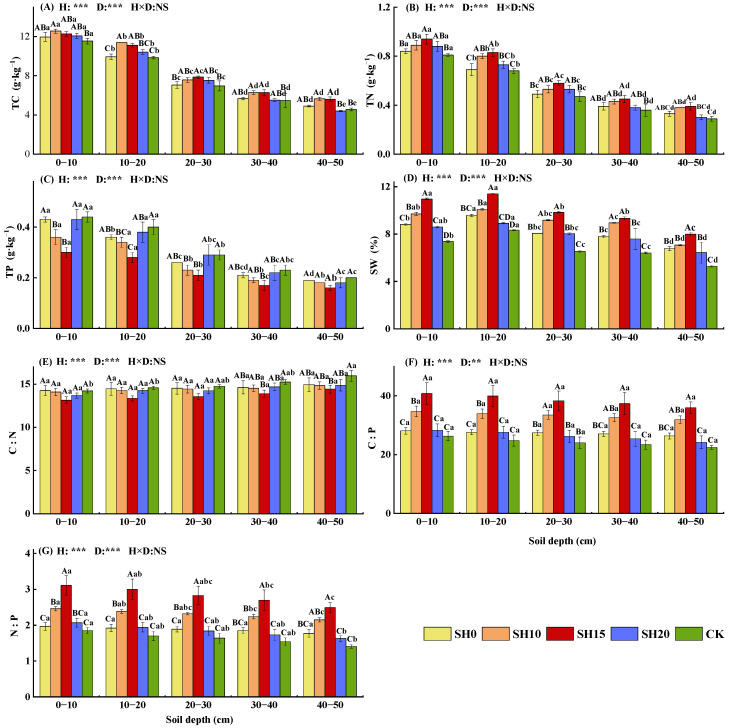
Effects of different stubble heights on soil characteristics in *H. rhamnoides* plantations (mean ± SE). (**A**) Total carbon; (**B**) total nitrogen; (**C**) total phosphorus; (**D**) soil water; (**E**) carbon-to-nitrogen ratio; (**F**) carbon-to-phosphorus ratio; (**G**) nitrogen-to-phosphorus ratio. Different capital letters indicate significant differences among different stubble patterns in the same soil layer (*p* < 0.05), whereas different lowercase letters indicate significant differences between different soil layers in the same stubble patterns (*p* < 0.05). **: *p* < 0.01, ***: *p* < 0.001, NS: not significant.

Two-way ANOVA showed that within the 0–50 cm soil layer, stubble treatment highly significantly increased soil TC, TN, carbon-to-phosphorus ratio (C:P), nitrogen-to-phosphorus ratio (N:P), and soil water content (SW) while highly significantly decreasing soil TP and carbon-to-nitrogen ratio (C:N) (*p* < 0.001) (Figure 2). The CV for soil characteristics among different stubble treatments ranged from 4.24% to 21.36%, with SW, TP, C:P, and N:P showing CV greater than 10% (Table 3). Among soil characteristics, C:P was the most sensitive to stubble treatment, with a CV of 21.36%. Significant differences were observed in soil TC, TN, TP, N:P, and SW across different soil layers (*p* < 0.001), whereas C:P showed highly significant differences (*p* < 0.01). The CV for soil nutrients across different layers ranged from 3.92% to 35.75%, with SW, TC, TN, and TP showing CV greater than 10%.

As soil depth increased, SW, TC, TN, TP, C:P, and N:P decreased. By contrast, soil C:N increased with depth. SW, TC, TN, C:P, and N:P initially increased and then decreased with increasing stubble height. TC reached its maximum value under SH10, whereas the other parameters peaked under SH15, Compared with CK, stubble treatment increased these parameters by 12.43% ± 6.07%, 8.20% ± 4.92%, 13.45% ± 6.86%, 29.58% ± 20.25%, and 36.12% ± 24.39%. However, TP and C:N significantly decreased (*p* < 0.05), with reductions of 14.36% ± 9.65% and 4.65% ± 2.30%, respectively, and followed the order of CK > SH20 > SH0 > SH10 > SH15.

### 2.3. Correlations Between Fine Roots and Soil Properties

**Figure 3:**
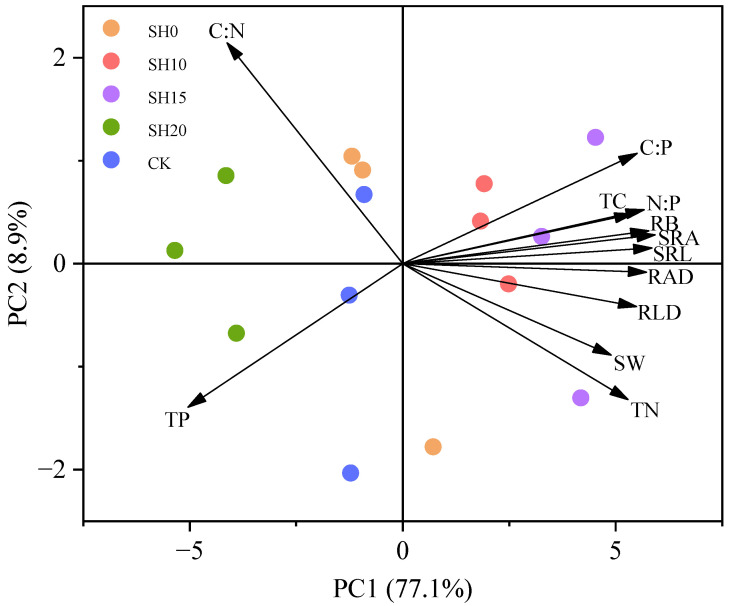
PCA of fine root traits and soil characteristics. RB: root biomass. RAD: root area density. RLD: root length density. SRA: specific root area. SRL: specific root length. TC: total carbon. TN: total nitrogen. TP: total phosphorus. SW: soil water content. C:N: carbon-to-nitrogen ratio. C:P: carbon-to-phosphorus ratio. N:P: nitrogen-to-phosphorus ratio.

PCA revealed changes in the fine root morphology of *H. rhamnoides* and soil characteristics after stubble treatments. The cumulative variance contribution rates of PC1 and PC2 exceeded 80% (Figure 3). Additionally, fine root and soil characteristics were significantly separated by stubble treatment along PC1. Fine root morphological indices, C:P, N:P, TC, and SW were closely associated with SH10 and SH15, whereas C:N and TP were closely associated with SH0, SH20, and CK. PCA revealed two dimensions of variations in fine root traits and soil characteristics with stubble height, namely, PC1 and PC2. The former promoted fine root growth and morphological characteristics of fine roots were primarily regulated by C:P, N:P, TC, and SW, whereas fine roots in the latter grew slowly, maintaining high soil TP content and C:N. These characteristics reflect the changes In the foraging strategies of *H. rhamnoides* plantations due to stubble treatment.

**Figure 4:**
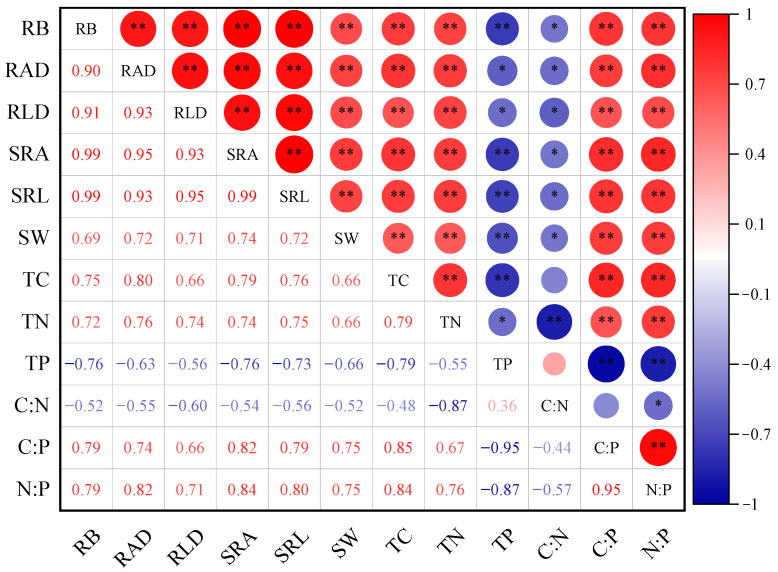
Correlation analysis between fine root traits and soil characteristics. RB: root biomass. RAD: root area density. RLD: root length density. SRA: specific root area. SRL: specific root length. TC: total carbon. TN: total nitrogen. TP: total phosphorus. SW: soil water content. C:N: carbon-to-nitrogen ratio. C:P: carbon-to-phosphorus ratio. N:P: nitrogen-to-phosphorus ratio.*: *p* < 0.05, **: *p* < 0.01.

In *H. rhamnoides* plantations, fine root morphological indices, namely, RB, RAD, RLD, SRA, and SRL, showed highly significant correlations with each other (*p* < 0.01) (Figure 4). The different morphological indices of fine roots exhibited a synchronized growth trend, with root surface area expansion being consistent with root length. SW, TC, TN, C:P, and N:P were significantly positively correlated with fine root morphological indices (*p* < 0.01), with N:P and C:P showing the highest correlations, which ranged from 0.71 to 0.84 and 0.66 to 0.82, respectively. TP and C:N were significantly negatively correlated with fine root morphological indices (*p* < 0.05). TC, TN, C:P, and N:P were significantly positively correlated with each other (*p* < 0.05) and negatively correlated with TP and C:N.

**Figure 5:**
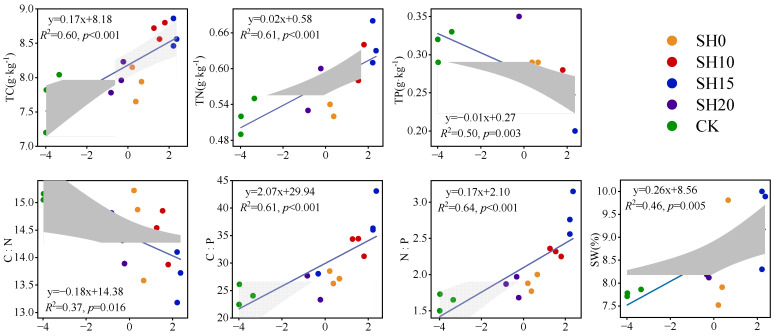
Relationship between PC1 of fine root traits and soil characteristics. PC1 of fine root traits represents the first axis after the PCA dimensionality reduction of fine root traits. TC: total carbon. TN: total nitrogen. TP: total phosphorus. SW: soil water content. C:N: carbon-to-nitrogen ratio. C:P: carbon-to-phosphorus ratio. N:P: nitrogen-to-phosphorus ratio.

Least squares linear regression revealed the response of fine root morphological traits (PC1 after PCA dimensionality reduction) to soil physicochemical properties (Figure 5). Fine root traits were positively correlated with TC, TN, C:P, N:P, and SW but were negatively correlated with TP and C:N. In particular, the soil nutrient quality indicators N:P and C:P explained 66% and 61% of the fine root morphological trait strategies during stubble treatment, respectively.

**Figure 6:**
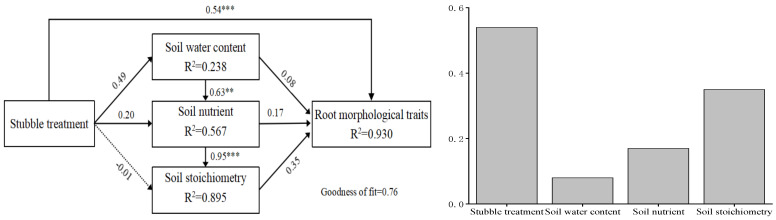
PLS-PM revealed the direct and indirect effects of stubble treatment, soil moisture, soil nutrients, and soil stoichiometry on the fine root traits of *H. rhamnoides*. Solid and dashed arrows represent positive and negative effects, respectively. The significance of standardized path coefficients is indicated by numbers on the arrows. (**, and *** denote significant differences at the *p* < 0.01, and *p* < 0.001 levels, respectively). R² represents the explained variance of individual variables. Treatments included stubble treatments with heights of 0, 10, 15, and 20 cm above ground level and non-stubble treatment.

PLS-PM was used to evaluate the direct and indirect effects of stubble treatment, soil nutrients, soil moisture, and soil stoichiometry on the morphological traits of fine roots in *H. rhamnoides* plantations (Figure 6). The model showed that stubble treatment and soil physicochemical properties explained 76% of the variation in fine root morphological traits. Each variable had a direct positive effect on the morphological traits of *H. rhamnoides* fine roots, with stubble treatment having the highest effect among variables (path coefficient of 0.54), followed by soil stoichiometry (path coefficient of 0.35). Stubble treatment had a positive effect on soil moisture (path coefficient of 0.49) and soil nutrients (path coefficient of 0.20) but a negative effect on soil stoichiometry (path coefficient of −0.01).

## 3. Materials and Methods

### 3.1. Overview of the Study Area

**Figure 7:**
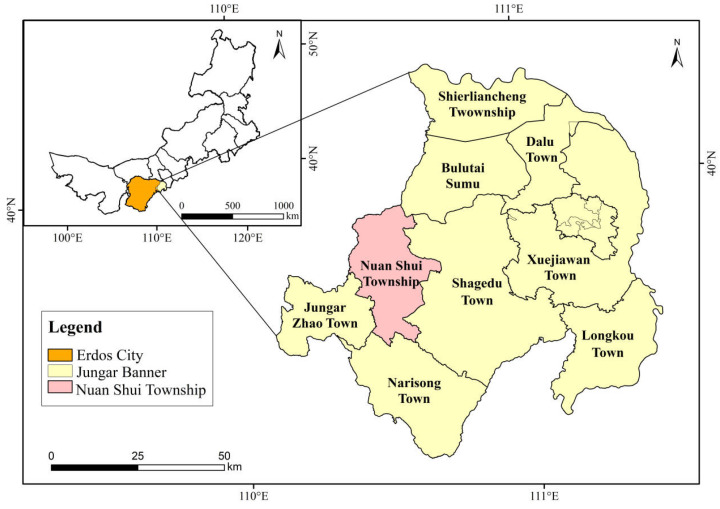
Location of the study area.

The study area is located in the Geqiu Gully watershed in the northern part of Nuanshui Township, Jungar Banner, Ordos City, Inner Mongolia Autonomous Region. Its geographical coordinates are E 110°25’–110°48’ and N 39°42’–39°50’ (Figure 7), covering an area of approximately 96 km^2^. In this region, the bedrock area is exposed, with the exposed area exceeding 70% of the total area. The overlying loess layer has a depth of 40–60 cm, and beneath it lies arsenic sandstone formed during the Paleozoic Permian, Mesozoic Triassic, Jurassic, and Cretaceous periods. The sandstone is mainly composed of interbedded sandstone, mudstone, and shale. The dominant soil type is chestnut calcareous soil. The topography is mostly characterized by ridge-like hills, with an average gully density of 5–7 km·km^−2^. The terrain is rugged, with numerous steep slopes, low diagenetic grade, and severe weathering of rock layers [30,31]. Its average elevation ranges from 800 m to 1590 m. The study area is in a typical mid-temperate semiarid continental monsoon climate zone. Its average annual sunshine duration is 2900–3100 h, with a frost-free period of 148 days. In the study area, the average annual precipitation is approximately 400 mm and the average annual evaporation is 2093 mm. The average annual temperature ranges from 6.2 °C to 8.7 °C. The annual total solar radiation is 143.4 kCal·m^−2^. Spring is characterized by strong and prolonged winds, with an average annual wind speed of 3.2 m·s^−1^ and a maximum wind speed of 32 m·s^−1^. The average annual number of windy days is 10–30. The watershed is primarily covered by artificial vegetation specifically planted for soil and water conservation, windbreaks, and sand fixation. The main afforestation species include *H. rhamnoides*, *Pinus tabuliformis*, *Caragana korshinskii*, *Medicago sativa*, and *Prunus sibirica* [32].

### 3.2. Site Selection

The experimental forest consists of *H. rhamnoides* plantations established in 2010. The establishment of *H. rhamnoides* forests used 2–3-year-old seedlings with a height of 40–50 cm and a diameter of 0.35–0.50 cm. A pit-type land preparation method, using dimensions of 0.5 m × 0.5 m × 0.4 m, was employed to preserve the original vegetation as much as possible and promote soil and water conservation. The experimental site includes four stubble treatment groups and one control group, with three replicate plots of 50 m × 50 m for each treatment, for a total of 15 replicate plots. Within the plots, *H. rhamnoides* trees were planted at a spacing of 2 m × 4 m. The following stubble treatments were applied to the plants in the plots before the thaw in March 2020: SH0—stubble treatment at 0 cm above ground level; SH10—stubble treatment at 10 cm above ground level; SH15—stubble treatment at 15 cm above ground level; SH20–stubble treatment at 20 cm above ground level; CK—non-stubble treatment. The stand conditions in the plots were re-evaluated in July 2021 after one year of natural recovery (Table 4).

### 3.3. Sample Collection

Within the standard plots, three standard clumps with similar growth conditions were selected through tree-by-tree measurement. Sampling was conducted on clear days without rainfall on the preceding seven days. Centered on the standard clumps, with a radius of one meter, surface litter was removed, and the 1/4-circle method was used to sample *H. rhamnoides* roots. In consideration of the environmental conditions of the standard clumps, their growth status, and data accuracy, a sector area perpendicular to the forest belt was selected as the data collection area, with a central angle of 90°. The selected sector area was vertically divided into five layers, each with a height of 10 cm. Root and soil samples were collected from each layer, and the collected roots and soil samples were brought back to the laboratory.

**Figure 8:**
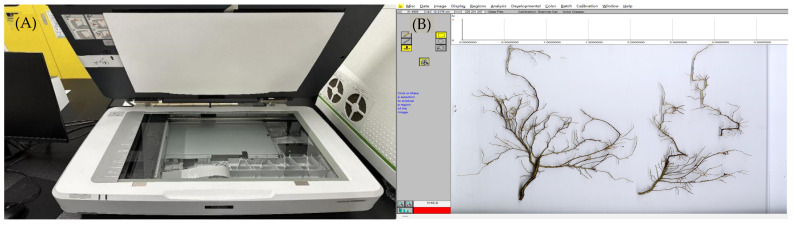
Fine root scanning and analysis. (**A**) Root scanner. (**B**) Root analysis.

The collected roots were placed in water and gently loosened to remove soil particles. Fine roots of *H. rhamnoides* with a diameter of less than 2 mm were carefully extracted by using tools, such as calipers and tweezers. Live roots were identified on the basis of their shape, color, elasticity, and ease of separation from the central root system [33]. The extracted fine roots were scanned by using an Expression 12000XL (Epson Corporation, Suwa, Nagano, Japan) [34] root scanner, and data on root length, surface area, and other parameters were obtained by using the WinRHIZO (Regent Instruments Inc., Quebec City, QC, Canada) [35] root analysis system (Figure 8). After scanning, the fine root samples were placed in an oven at 80 °C and dried to a constant weight. The dry mass of the fine roots was measured by using an electronic balance, and fine RB, root area density (RAD), root length density (RLD), specific root area (SRA), and SRL were calculated. Soil total nitrogen (TN) and total carbon (TC) content were measured with a Vario EL elemental analyzer [36]. Soil total phosphorus (TP) was determined through the molybdenum–antimony anticolorimetric method [37], and soil water content (SW) was measured by using the drying method [38].

The formulas for calculating fine root morphological and soil water content parameters are as follows [39]:

RB (g·m^2^) = RDW (g)/S (m^2^)

RAD (cm^2^·m^3^) = RA (m^2^)/V (m^3^)

RLD (m·m^3^) = RL (m)/V (m^3^)

SRA (cm^2^·g^−1^) = RA (cm)/RDW (g)

SRL (m·g^−1^) = RL (m)/RDW (g)

SW(%) = {W_1_[m] − W_2_[m]}/{W_2_[m] − W[m]} × 100%

In these formulas, RB is the root biomass, RDW is the root dry weight, and S is the soil cross-section area. Moreover, RAD is the root area density, RA is the root area, V is the soil volume, RLD is the root length density, and RL is the root length. Furthermore, SRA is the specific root area, SRL is the specific root length, and SW is the soil water content. Finally, W_1_ is the total weight of the wet soil and aluminum box, W_2_ is the total weight of the dry soil and aluminum box, and W is the weight of the aluminum box.

### 3.4. Data Analysis

One-way ANOVA was used for the statistical analysis of the differences in fine root traits and soil properties between different stubble treatments and soil layers. Two-way ANOVA was employed to examine the interaction effects between treatments and soil layers. Fisher’s least significant difference method was applied to test significance, with a significance level of *p* < 0.05. Principal component analysis (PCA), Pearson correlation analysis, and linear regression analysis were conducted to explore the relationships between fine root traits and soil characteristics. Data analysis was performed with DPS 9.01 software, and graphs were created by employing Origin 9.4. The partial least squares path model (PLS-PM) [40] was implemented by applying the “plspm” package in R version 4.2.1 (R Development Core Team, 2022) to evaluate the potential pathways, including stubble treatment, soil moisture, nutrients, and stoichiometry, influencing fine root morphological traits.

## 4. Discussion

### 4.1. Effects of Stubble Treatment on Fine Root Morphological Traits

Root traits determine the absorption of nutrients and water essential for plant survival and growth and reflect the extent to which plants respond to environmental changes. In trees, stubble treatment weakens the dominance of aboveground growth, enhances sprouting capacity, and induces the formation of a fine root system with high plasticity in response to soil characteristics, thereby increasing resource allocation to belowground components [41,42]. Some studies have shown that [43] plant RB decreases after cutting or harvesting due to reduced aboveground leaf area. However, numerous works have demonstrated that cutting or harvesting increases RB [44,45]. Bond [46] suggested that plants undergo compensatory growth after aboveground damage to promote regeneration. In this study, fine root traits under ground-level stubble treatment were clearly separated from those under non-stubble treatment. The RB of *H. rhamnoides* initially increased and then decreased with increasing stubble height, reaching its maximum value of 320.46 g·m^−2^ under SH15. This result was likely obtained because under conditions wherein severe damage and soil erosion are avoided, the selection of an appropriate stubble height can improve light, water, heat, and nutrient conditions in forests [47]. This effect enhances the photosynthetic efficiency of trees, leading to the increased allocation of photosynthetic products to the root system, effectively compensating for the fine root mortality caused by low stand density [48]. Additionally, *H. rhamnoides* is a typical clonal plant with strong root sprouting and spreading potential, and suitable stubble heights remarkably promote fine root growth [49]. Compared with those under CK, the proportion of RB in the topsoil decreased, whereas the proportion of RB in the 30–50 cm soil layer gradually increased under stubble treatment. The RB proportion under SH15 was 1.90 times that under CK, revealing a mechanism by which the stubble treatment promotes RB investment and allocation. In alignment with the findings of You [50], an appropriate stubble height can encourage the allocation of RB to deep soil layers, thus reducing competition for resources in the topsoil.

RLD and RAD are direct indicators of soil space exploration, whereas SRL and SRA are important metrics for assessing the nutrient absorption efficiency of plant fine roots [51,52,53]. The present study shows that the RLD, RAD, SRL, and SRA of *H. rhamnoides* initially increased and then decreased with increasing stubble height, peaking under SH15 with values of 148.18 m·m^−3^, 34.36 cm·m^−3^, 341.89 m·g^−1^, and 13.25 cm²·g^−1^, respectively. RLD, RAD, SRL, and SRA under SH15 were significantly different from those under CK (*p* < 0.05). RLD and RAD in the 30–40 and 40–50 cm soil layers were 2.51 and 4.97 times higher under SH15, respectively, than under CK. The trends of RLD and RAD with stubble height were largely consistent with those of RB, indicating the strong influence of RB per unit soil volume. Correlation analysis revealed highly significant relationships among fine root morphological indices after stubble treatment (*p* < 0.01), with root absorption area and length showing synchronized growth, suggesting the coevolution of the foraging efficiency of fine roots. PCA results indicated that fine root traits in *H. rhamnoides* after stubble treatment centered on high SRA (0.316) + SRL (0.312) as the core strategy, maximizing soil resource capture by increasing the absorption area and length per unit root mass. The high weights of RB (0.308) and RAD (0.305) confirmed that stubble treatment is a direct driver of fine root regeneration, allocating a substantial portion of photosynthetic products to belowground parts. The higher weights of SRA and SRL than those of RAD, RLD, and RB also indicated that fine roots adopt a resource-acquisition ecological strategy prioritizing morphological efficiency over length expansion after stubble pruning, with SH15 demonstrating the highest exploration–absorption synergy among treatments.

### 4.2. Effects of Stubble Treatment on Soil Characteristics in Forest Plantations

An appropriate stubble height not only promotes the growth of fine roots in forest trees but also accelerates soil improvement, enhancing soil moisture and fertility [54]. Studies have shown that [55,56] stubble treatment substantially reduces water consumption by trees and improves soil moisture conditions. The present work found that SW in *H. rhamnoides* plantations initially increased and then decreased with increasing stubble height and reached its highest value of 9.40% under SH15. SW under SH15 was 1.21 times that under CK. Wei [57], through research on the effects of stubble treatment on the vegetation characteristics and soil physicochemical properties of *Hedysarum scoparium* shelterbelts, found that appropriate stubble treatment positively affected forest habitats. However, during the rapid recovery phase after stubble treatment, the ground surface should be covered to prevent water loss. Chen [58] analyzed soil moisture conditions in *H. rhamnoides* plantations after stubble treatment and found that the soil moisture recovery capacity was remarkably enhanced. This finding is consistent with the results of the present study. It may be attributed to the reduction in water consumption by aboveground plant tissues caused by stubble treatment, allowing water to accumulate in the soil. Additionally, stubble treatment promotes root sprouting, and the ability of roots to retain water and stabilize soil further enhances soil moisture recovery [59].

Soil nutrients are the material basis of fertile soil, and their abundance and forms directly affect soil fertility and indirectly influence forest health [60,61,62]. Tree branches contain considerable amounts of nutrients and cellulose. After stubble treatment, the residual branches and fallen leaves of *H. rhamnoides* return to the soil, increasing organic matter input and promoting nitrogen fixation and phosphorus cycling [63]. This work found that soil TC and TN content initially increased and then decreased with increasing stubble height, peaking at 8.69 and 0.64, respectively, under SH15. Soil TC and TN contents under SH15 were 1.13 and 1.23 times higher than those under CK. Consistent with this study, the work of Bian [64] analyzed the effects of stubble treatment on soil nutrient content and confirmed that stubble treatment increases soil TC and TN content while positively improving soil physical properties. The substantial addition of organic matter from residual branches and leaves, combined with increased root exudates during the compensatory recovery phase, leads to high soil TC content [65,66,67]. The soil TN content of *H. rhamnoides*, a typical nitrogen-fixing plant, primarily depends on the nitrogen-fixing capacity of root nodules. Stubble treatment enhances the activity of nitrogen-fixing bacteria, increasing soil nitrogen content [68,69]. By contrast, soil TP content decreased after stubble treatment. In arsenic sandstone areas, phosphorus, as a limiting element, mainly originates from rock weathering and leaching. After stubble treatment, organic acids produced by the decomposition of fallen branches and leaves can bind with metal ions in the soil, breaking phosphorus limitations and releasing fixed phosphorus. Additionally, during the compensatory growth phase, the increased demand of plants for phosphorus leads to the active absorption of soil phosphorus by roots, resulting in remarkable reductions in soil phosphorus content [70]. The carbon-to-nitrogen-to-phosphorus ratio is the mass ratio of carbon, nitrogen, and phosphorus in soil organic matter or other components. It is an important parameter for determining the balance of soil carbon, nitrogen, and phosphorus and a key indicator of soil organic matter composition and nutrient balance [71,72]. After stubble treatment, soil C:N significantly decreased and remained relatively stable because *H. rhamnoides* is a nitrogen-fixing plant and its roots host numerous nitrogen-fixing bacteria, leading to a higher increase in soil nitrogen content than in carbon content. The increase in C:P and N:P is due to the increased demand for phosphorus during the compensatory growth phase [73]. This study found that the equilibrium points of soil C:N, C:P, and N:P under different stubble heights all occurred under SH15, indicating that among treatments, SH15 is most conducive to maintaining soil nutrient balance and effectively improving soil quality and fertility.

### 4.3. Stubble Treatment Drives the Coevolution of Fine Roots and Soil

As a disturbance measure, stubble disrupts the aboveground–underground resource balance, eliminates apical dominance, stimulates the compensatory growth of fine roots, and reconstructs the water and nutrient absorption system [74]. PLS analysis indicated that stubble had a direct positive effect on fine roots, with the direct effect being dominant (path coefficient of 0.54) and higher than the sum of all soil-mediated effects, proving that stubble is the direct driving force behind the morphological regeneration of *H. rhamnoides* fine roots. Although stubble had a direct positive effect on SW (path coefficient of 0.49), the direct positive effect of SW on fine root morphology was relatively low (path coefficient of 0.08) due to the specific water-saving strategies of fine roots in arid regions. However, SW has a pivotal role and crucial marginal effects. An increase in SW can promote mineralization, converting organic phosphorus in residual litter into available forms after stubble treatment, acting as a switch variable for nutrient availability. Therefore, the indirect effects of SW reinforce the role of water as the primary limiting factor in arid regions [75]. Stubble positively affected soil nutrients (path coefficient of 0.20) likely due to the turnover of litter and fine roots, indicating that the accumulation of TC and TN exceeded the loss of TP. Soil nutrients had a positive effect on fine roots (path coefficient of 0.17), signifying that after stubble treatment, soil nutrients improved in terms of quality rather than increasing in terms of total quantity. The mineralization effect of soil moisture, combined with the organic acids produced by the decomposition of litter, activates previously unavailable phosphorus [76]. Simultaneously, changes in fine root morphology increases the contact area between fine roots and soil, allowing for the preferential interception and absorption of activated available phosphorus, thereby breaking the phosphorus limitation in arsenic sandstone areas. Stubble can enhance the nitrogen-fixing activity of rhizobia, thus promoting the fixation of nitrogen in the soil. Additionally, synergistic carbon fixation from the turnover of litter and fine roots after stubble treatment forms a collaborative vegetation–soil restoration mechanism in arid regions. The breaking of phosphorus limitation, fixation of nitrogen, and sequestration of carbon collectively optimize soil nutrient quality after stubble treatment. The indirect effects of stubble treatment through water content (0.49)–nutrients (0.63)–stoichiometry (0.95) were far greater than the direct effect on stoichiometry (−0.01), indicating that water content plays a key role in soil nutrient mineralization and breaking phosphorus limitation. Soil stoichiometry had a positive effect on *H. rhamnoides* (path coefficient of 0.35) that was greater than the direct effect of nutrients on fine roots (path coefficient of 0.17). The high weights of N:P and C:P in PCA indicated that stoichiometry dominated the nutrient response, with N:P and C:P exhibiting highly significant positive correlations with the SRA of fine roots (*p* < 0.01). Least squares linear regression indicated that soil nutrient quality in terms of N:P and C:P can explain 66% and 61% of the root morphological trait strategies during stubble treatment, respectively, demonstrating that fine roots exhibit high adaptability to the breaking of phosphorus limitation and fixation of carbon and nitrogen after stubble treatment. Changes in the ratio of phosphorus to other elements in the soil play a key role in shaping fine root morphology. In arsenic sandstone areas, where phosphorus limitation inherently exists [77], stubble promotes the transformation of inert phosphorus pools into active phosphorus sources through soil water mineralization and root remodeling, altering the pattern of soil nutrient supplies. Fine roots adopt a foraging + absorption functional coevolution strategy centered on high SRL and SRA to cope with environmental changes. This research result shows that by selecting an appropriate stubble height, a positive cycle of nitrogen fixation–phosphorus release–carbon sequestration–root promotion can be constructed, hence providing a scientific theoretical basis for ecological restoration in arsenic sandstone areas.

## 5. Conclusions

This study demonstrates that the RB, RAD, RLD, SRA, and SRL of *H. rhamnoides* initially increase and then decrease with increasing stubble height, reaching their peak values under a stubble height of 15 cm, exhibiting significant differences (*p* < 0.05). Furthermore, stubble alters the vertical distribution of *H. rhamnoides* fine roots, increasing the proportion of fine roots in the 30–50 cm soil layer. PLS analysis reveals that stubble has a direct positive effect on fine roots, with the direct effect being dominant (path coefficient of 0.54) and higher than the sum of all soil-mediated effects. After stubble treatment, the morphological indices of *H. rhamnoides* fine roots exhibit highly significant correlations (*p* < 0.01), indicating the synchronous growth of absorption area and length. PCA results indicate that after stubble treatment, the fine root traits of *H. rhamnoides* adopt a core strategy of high SRA (0.316) + high SRL (0.312). By increasing the absorption area and length per unit mass of roots, *H. rhamnoides* maximizes the capture of soil resources, shifting toward an ecological strategy wherein morphological efficiency takes precedence over length expansion in arid areas.

SW, TC, TN, C:P, and N:P all initially increase and then decrease with increasing stubble height, reaching their highest values under a stubble height of 15 cm, showing significant differences (*p* < 0.05). PLS analysis indicates that although stubble has a direct positive effect on SW (path coefficient of 0.49), the direct positive effect of SW on fine root morphology is low (path coefficient of 0.08). However, the indirect effects of stubble through SW (0.49)–nutrients (0.63)–stoichiometry (0.95) are far greater than the direct effect of stoichiometry (−0.01). The increase in soil moisture promotes soil mineralization, acting as a switch variable for nutrient availability. Therefore, the indirect effects of soil moisture reinforce the role of water as the primary limiting factor in arid regions. Least squares linear regression demonstrates that soil nutrient quality in terms of N:P and C:P can explain 66% and 61% of the root morphological trait strategies during stubble treatment, respectively. This finding indicates that after stubble treatment, fine roots exhibit high adaptability to the breaking of phosphorus limitation and fixation of carbon and nitrogen, with the changes in the ratio of phosphorus to other elements in the soil playing a key role in shaping fine root morphology.

The findings of this study indicate that the 15 cm stubble treatment for *H. rhamnoides* in the studied arsenic sandstone area results in fine roots exhibiting optimal foraging–absorption synergy and nutrient utilization efficiency. This treatment establishes the most rational positive cycle of nitrogen fixation, phosphorus release, carbon sequestration, and root promotion, providing a scientific theoretical basis for ecological restoration in arsenic sandstone areas.

## Figures and Tables

**Table 1:** Effects of different stubble heights on the fine root biomass of *H. rhamnoides*.

Stubble Treatment	Soil Layer (cm)					Total Biomass (g·m^−2^)
	0–10	10–20	20–30	30–40	40–50	
SH0	74.74 ± 0.70 Bb	96.11 ± 2.13 Ba	47.98 ± 1.32 Bc	40.76 ± 0.61 Cd	27.82 ± 0.85 Ce	287.41 ± 4.39 C
SH10	77.38 ± 0.94 ABb	99.60 ± 2.26 ABa	51.88 ± 0.52 Ac	45.14 ± 0.66 Bd	30.39 ± 0.60 Be	304.39 ± 4.32 B
SH15	80.73 ± 2.24 Ab	103.13 ± 1.17 Aa	55.43 ± 0.92 Ac	48.57 ± 0.49 Ad	32.60 ± 0.41 Ae	320.46 ± 0.63 A
SH20	66.44 ± 1.02 Cb	85.32 ± 0.41 Ca	43.28 ± 1.14 Cc	35.36 ± 0.26 Dd	23.52 ± 0.46 De	253.92 ± 2.36 D
CK	51.86 ± 0.19 Db	68.24 ± 0.44 Da	28.30 ± 1.50 Dc	26.45 ± 0.18 Ec	16.30 ± 0.20 Ed	191.16 ± 2.01 E
Stubble heights (H): *** Soil depth (D): *** H × D: ***						

Notes: Different capital letters indicate significant differences between different stubble patterns in the same soil layer, whereas different lowercase letters indicate significant differences between different soil layers in the same stubble patterns. ***: *p* < 0.001.

**Table 2:** Morphological characteristics of the fine roots of *H. rhamnoides*.

Traits	Traits	Sample Size	Minimum Value	Maximum Value	Mean Value	Coefficient of Variation Among Stubble Treatments	Coefficient of Variation Among Soil Layers
Root biomass (g·m^2^)	RB	75	16.05	104.75	54.29	16.92%	42.52%
Root area density (cm^2^·m^−3^)	RAD	75	71.38	1138.09	457.10	21.64%	53.44%
Root length density (m^2^·m^−3^)	RLD	75	16.40	272.41	121.052	20.33%	58.17%
Specific root area (cm^2^·g^−1^)	SRA	75	6.87	17.41	12.36	7.16%	15.98%
Specific root length (m·g^−1^)	SRL	75	157.60	419.65	320.20	6.36%	21.56%

**Table 3:** Soil characteristics of *H. rhamnoides* plantations.

Traits	Traits	Sample Size	Minimum Value	Maximum Value	Mean Value	Coefficient of Variation Among Stubble Treatments	Coefficient of Variation Among Soil Layers
Soil water (%)	SM	75	6.09	11.24	8.56	12.66%	12.09%
Total carbon (g·kg^−1^)	TC	75	4.31	12.83	8.18	5.61%	33.29%
Total nitrogen (g·kg^−1^)	TN	75	0.27	0.99	0.58	8.66%	35.75%
Total phosphorus (g·kg^−1^)	TP	75	0.14	0.49	0.28	13.57%	30.20%
Carbon-to-nitrogen ratio	C:N	75	12.61	16.64	14.38	4.24%	3.92%
Carbon-to-phosphorus ratio	C:P	75	29.94	45.96	29.94	18.76%	6.55%
Nitrogen-to-phosphorus ratio	N:P	75	1.35	3.48	2.10	21.36%	7.97%

**Table 4:** Basic information of the sampling plots.

Stubble Treatment	Sample Information				Stand Factor			
	Age (yr)	Size of Plots (m × m)	Slope (°)	Slope Position	Average Plant Height (cm)	Average Crown Width (cm) EW/SN	Current Year Branch Length (cm)	Current Year Basal Diameter (cm)
SH0	11	50 × 50	4	Upper slope	59.37	43.75/41.54	103.54	1.71
SH10	11	50 × 50	4	Upper slope	65.53	51.93/48.77	116.24	1.83
SH15	11	50 × 50	4	Upper slope	72.46	54.72/52.77	127.42	1.90
SH20	11	50 × 50	4	Upper slope	62.35	46.77/43.74	99.87	1.65
CK	11	50 × 50	4	Upper slope	103.54	89.85/81.78	64.93	1.21

Note: SH0: stubble treatment at 0 cm above ground level, SH10: stubble treatment at 10 cm above ground level, SH15: stubble treatment at 15 cm above ground level, SH20: stubble treatment at 20 cm above ground level; CK: non-stubble treatment.

## Data Availability

The original contributions presented in the study are included in the article. Further inquiries can be directed to the corresponding author.
